# Increasing Level of Leisure Physical Activity Could Reduce the Risk of Hip Fracture in Older Women: A Dose–Response Meta-analysis of Prospective Cohort Studies

**DOI:** 10.1097/MD.0000000000002984

**Published:** 2016-03-18

**Authors:** Ke Rong, Xiao-yu Liu, Xu-hua Wu, Xiao-liu Li, Qing-quan Xia, Jiong Chen, Xiao-fan Yin

**Affiliations:** From the Department of Orthopedics (KR, XHW, QX, JC, XFY), Minhang Hospital, Fudan University, Shanghai, China; Shanghai Institute of Medical Imaging (XYL), Shanghai, China; Department of Interventional Radiology (XYL), Zhongshan Hospital, Fudan University, Shanghai, China; Department of Epidemiology (XYL), School of Public Health, Fudan University, Shanghai, China; and Department of Rehabilitation Medicine (XL), Minhang Hospital, Fudan University, Shanghai, China.

## Abstract

We carried out the study to investigate and quantitatively assess the potential association between current level of physical activity and the risk of osteoporosis hip fracture in older women.

Relevant publications before October 2015 were identified using the PubMed and Ovid searching tools. A dose–response meta-analysis was carried out to combine and analysis results. Fourteen prospective studies were included in the meta-analysis. A general analysis of 9 studies showed a significant inverse relationship between increasing level of physical activity and risk of hip fracture in older women [relative risk (RR) = 0.93, 95% confidence interval (95% CI): 0.91–0.96]. The result of a sensitivity analysis was consistent with the general analysis (RR = 0.94, 95% CI: 0.93–0.96). The association between increasing level of physical activity and risk of wrist fracture was not statistically significant in a general analysis of three studies (RR = 1.004, 95% CI: 0.98–1.03). A potential direct association between increasing level of physical activity and risk of wrist fracture was observed after removing 1 study with the greatest weight (RR = 1.01, 95% CI: 1.00–1.03). No significant publication bias was observed in our analysis.

Our results show that increasing level of physical activity within an appropriate range may reduce the risk of hip fracture but not the risk of wrist fracture in older women.

## Abstract 2

Supplemental Digital Content is available in the text

## INTRODUCTION

The occurrence of fragility fractures among older adults is an important public health concern because of the associated high morbidity and heavy social burden.^1–3^ The incidence and medical expense is predicted to increase rapidly over the coming decades.^4^ Moreover, population aging is likely to further escalate this condition. Women aged 45 years or older are considered more vulnerable to fragility fractures due to the potential osteoporosis and obesity after menopause.^5,6^ Therefore, the prediction and prevention of fragility fractures is a particular priority for older women.

Physical activity is a simple and inexpensive way to gain health. The American Center for Disease Control and Prevention has recommended that individuals undertake a regime of aerobic and muscle-strengthening physical activity.^7,8^ Physical activity is also a therapeutic tool against pre- and postmenopausal loss of bone density. In recent decades, the association between physical activity and bone health in older adults has aroused particular attention. It is reported that bone mineral density (BMD) could be improved through an appropriate amount of physical activity in the older population. In the Osteoporosis Risk Factor and Prevention Study, Rikkonen et al^9^ found that regular physical activity significantly decreased proximal femur bone loss among older women after 15 years of follow-up. In addition, the results of several meta-analyses have also shown that BMD could benefit from physical activity.^10^ However, long-term weight-bearing or excessive exercise may lead to stress fractures, especially in older women with osteoporosis.^5,11^ Several prospective studies have investigated the association between physical activity and the risk of fracture in older women. However, the relationship between physical activity and the risk of fragility fracture varies according to level of physical activity and fracture site.^12–16^

We conducted a dose–response meta-analysis to assess the association between level of physical activity and risk of fracture in older women. A subgroup analysis was also performed to investigate the relationship between level of physical activity and risk of fracture in a predetermined site.

## METHODS

### Literature Search

We carried out a literature search using the PubMed (MEDLINE) and Ovid (Embase and Cochrane library) searching tools. The search was restricted to prospective cohort studies investigating the association between physical activity and risk of fracture in older women, published between January 1990 and October 2015. The following key words and heading terms were used in the PubMed search: “(bone fracture) AND ((physical activity) OR exercise) AND women.” Similar heading terms were used in the Ovid search, with restrictions to the Embase and Cochrane Library. Furthermore, reference lists of all included studies were scrutinized. We followed the standard criteria for conducting and reporting a meta-analysis of observational studies.^17^ All analyses were based on previous published studies; thus, patient consent and ethical approval were not required.

### Study Selection Criteria

Studies were included if they met the following criteria: prospective cohort study; investigated the association between physical activity and risk of fracture in older women; size effect evaluated using relative risk (RR), odds ratio (OR), or hazard ratio (HR) values with 95% confidence intervals (CIs), or sufficient data provided to calculate these values; and physical activity divided into at least 3 quantitative categories. In addition, studies conducted in a population with exposure to chronic disease (excluding osteoporosis) or drug consumption were excluded. If several studies were conducted in the same population, publications with the most relevant information and longest period of follow-up were included. The final list of included studies was obtained from the combined searches and selections of 2 independent investigators (KR and XFY).

### Data Extraction and Quality Assessment

Data were extracted independently by 2 investigators (KR and XYL using a standard-format table). The following information was extracted from each study: author's name, publication year, published journal, study design, geographic region, age at baseline, level of physical activity, sample size (women only), number of years of follow-up, adjusted covariates, endpoint outcomes, endpoint case ascertainment, and RR, HR, or OR with 95% CIs for each physical-activity level. The number of person-years was extracted directly or calculated using the follow-up time provided and the number of participants quoted in the publication.

According to the Newcastle–Ottawa Scale,^18^ a 12-score grading system was used in the quality assessment, principally across the following 4 domains: selection of participants; exposure measurement; study design and control of potential bias; and comprehensive assessment of outcomes and follow-up. Studies scored greater than 9 were considered to be of “good” quality in our meta-analysis. RK and XFY evaluated the quality score independently, and the mean value was used as the final score.

### Statistical Analysis

RRs of the included studies were used as the effect size in the meta-analysis. Given that the absolute risk of fragility fracture is relatively low in older women,^12,19,20^ HRs and ORs were deemed equivalent to RRs in the analysis. RRs, HRs, or ORs were standardized using the effective count method proposed by Hamling et al^21^ to make the group with the lowest physical-activity level the reference group in each study. Women-specific RRs were extracted from general searches.^12,16,22–25^ For publications that did not present stratified RRs in the female population, a binary logistic model was used to estimate stratified RRs using the fracture case and person-years.

For each study included, we calculated the study-specific slopes across increasing physical-activity level with 95% CIs using a liner regression model proposed by Greenland and Longnecker.^26^ We derived the RR value for every 3 units of increased physical activity for risk of fracture based on the liner model. The forest plot and analysis of publication bias was based on the derived RRs.

Potential heterogeneity was estimated using the Cochran Q and I^2^ statistics. A *P* value ≤0.1 was considered to indicate significant heterogeneity in the Q-test. The I^2^ statistics, which represent the total variation contributed by included studies, was used to assist the heterogeneity estimation. I^2^ > 75% was considered to indicate significant heterogeneity. A fixed-effect model (Mantel–Haenszel method) was used when the heterogeneity was not considered to be significant; otherwise, the random-effect model (DerSimonian–Laird method) was used in the analysis.

Publication bias was evaluated using the Begg test and Egger linear regression.^27^ A 2-sided *P*-value <0.05 was considered to be statistically significant. A funnel plot was presented in the final analysis.

## RESULTS

### Literature Search

**FIGURE 1:**
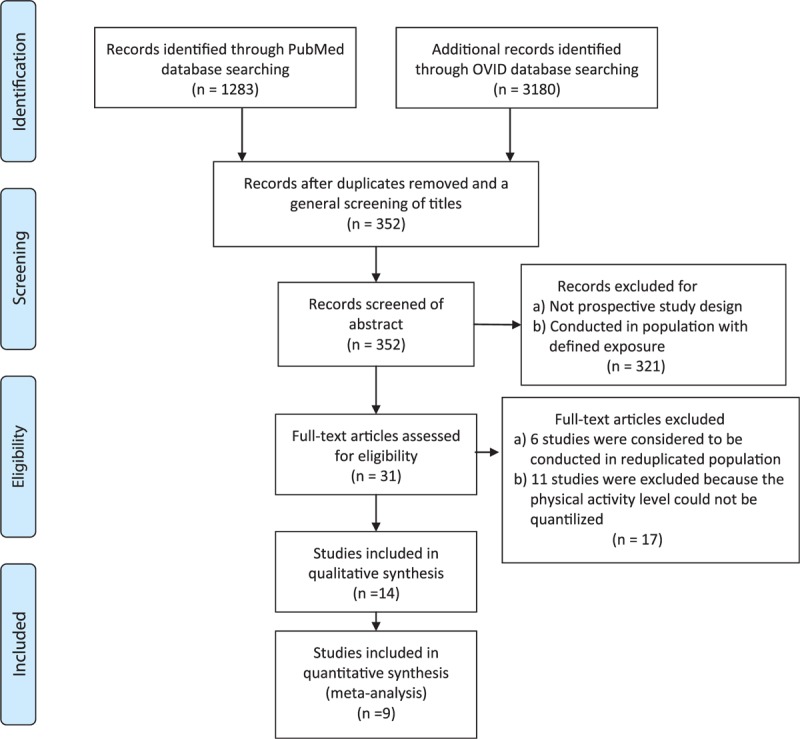
Selection of studies for inclusion in the dose–response meta-analysis.

Figure 1 shows the process of literature search and selection using a flow chart. We identified 1273 potential articles in PubMed and 3180 articles in Ovid (Embase and Cochran library) published between January 1990 and October 2015. After removal of duplicates and screening of the general criteria, 352 articles remained for further review. A total of 321 papers were excluded after a review of the abstract due to a small sample size, retrospective study design, or exposure to chronic disease and medicine, leaving 31 potential articles for full-text review. After a full-text evaluation of the 31 articles, 14 papers were finally included in our meta-analysis. Six papers were excluded as repeated reports of the same population and 11 papers were excluded due to lack of sufficient data or for not reporting the RR for men and women separately.

### Study Characteristics and Quality Assessment

Tables 1 and 2 present the characteristics and data extracted from the 14 included studies, 13 of which were of prospective cohort design and 1 that was considered to be a prospective nested case–control study. Data from 9 studies were finally analyzed in the dose–response meta-analysis. Data from the rest 5 papers are presented in Tables 1 and 2 but not included in the final analyses, because the investigated fracture sites were rarely reported in other literature and the information was insufficient for combined analysis. All included articles were published from 2002 to 2012, including 28,871 fracture cases and 1,380,285 participants (women only). The total observation time was estimated to be 12,127,159 person-years. Hip fracture and wrist fracture were 2 most common fractures in the cohort follow-up. Nine thousand four hundred one hip fractures and 10,960 cases were observed in the general studies. Physical activity was predominantly measured using a self-administered questionnaire, and the endpoint of fractures was confirmed by self-report diagnosis or medical record. Of the 14 included prospective studies, 3 were conducted in the United States and the other 11 were conducted among European countries (4 in the UK, 2 in Finland, 1 in Sweden, 1 in Norway, and 1 in Denmark and 1 in France). Quality assessments (score 1–12) are listed in Table 1. Twelve included studies were considered to be of “good” quality (Quality Score> = 9) and 2 were considered to be of “intermediate” quality (Quality Score <9 and ≥6) for our meta-analysis.

**TABLE 1:**
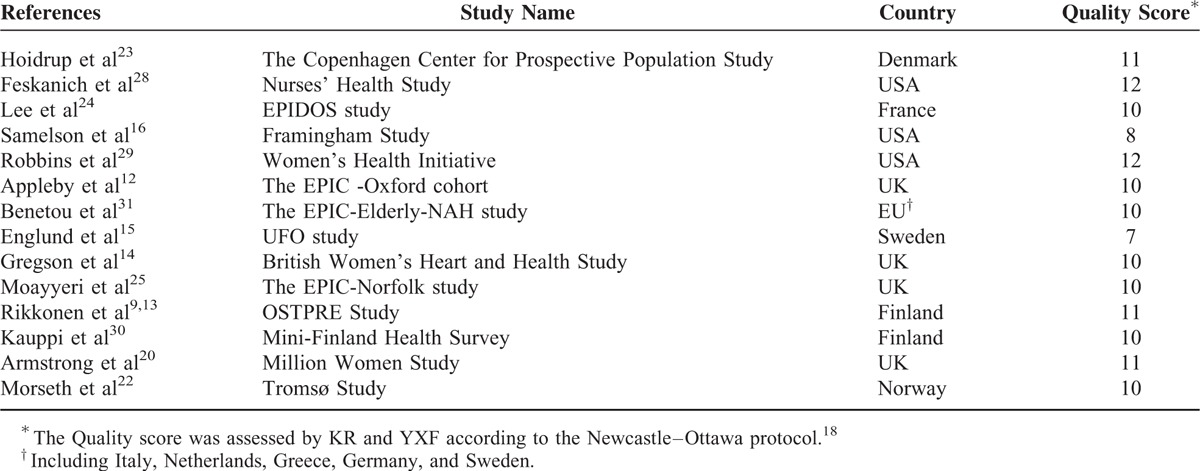
Characteristics of the Included Studies of Physical-activity Level in Relation to Risk of Fracture in Older Women

**TABLE 2:**
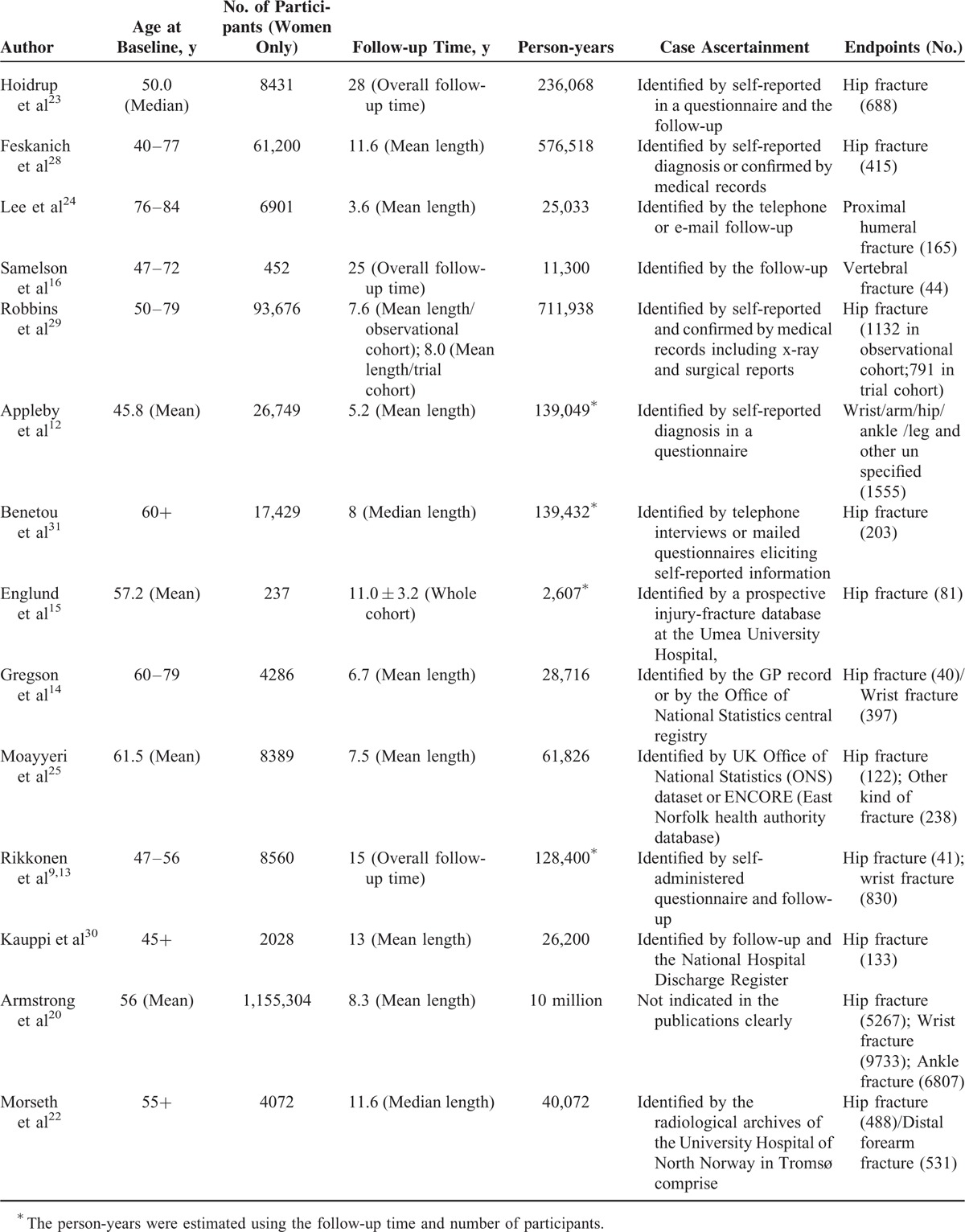
Follow-up Information of the Included Studies of Physical-activity Level in Relation to Risk of Fracture in Older Women

### Association Between Physical Activity and the Risk of Hip Fracture in Older Women

**FIGURE 2:**
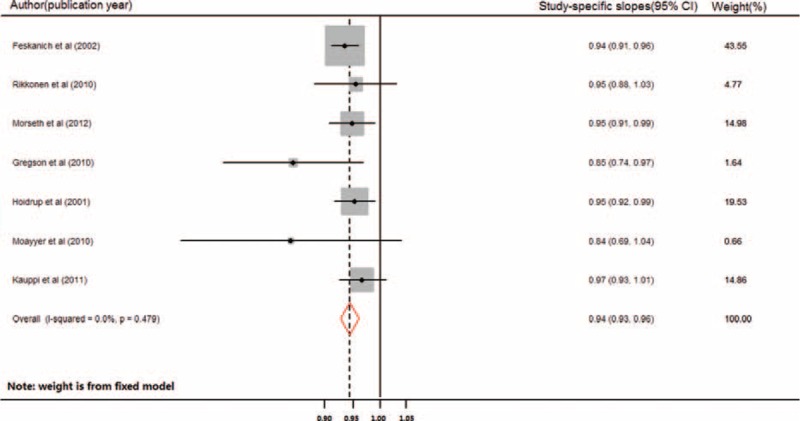
Forest plot (fixed-effect model) of increasing level of physical activity and risk of hip fracture in older women. The horizontal line indicates the study-specific 95% confidence interval. The square indicates the study-specific weight from the fixed-effect analysis. The diamond indicates the combined relative risk of the 7 included studies after the sensitivity analysis.

**FIGURE 3:**
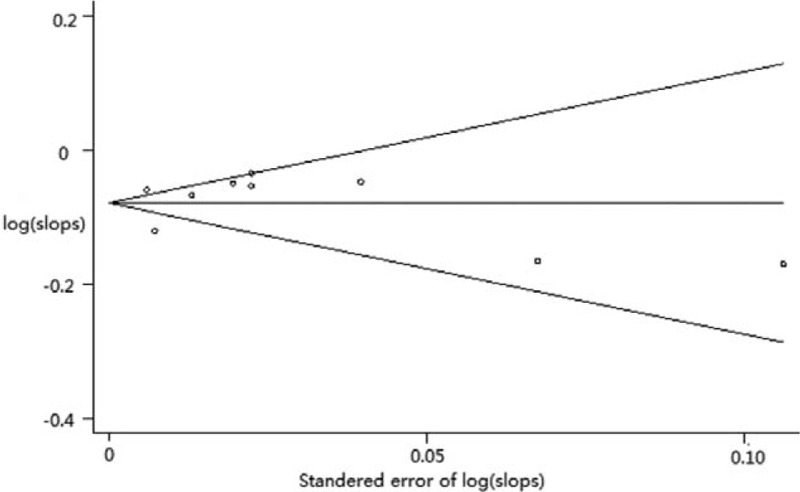
Funnel plot (Begg test) of 9 studies included in the analysis of association between increasing level of physical activity and hip fracture in older women (SE: standard error).

Nine studies were included in the dose–response meta-analysis for hip fracture.^13,14,20,22,23,25,28–30^ The combined RR for every increase of 3 physical-activity units in each study was 0.93 (95% CI: 0.91–0.96, S1 Table). Significant heterogeneity was observed between these studies (Q = 54.19, *P* < 0.0001; I^2^ = 85.2%). In the sensitivity analysis, 2 studies^20,29^ with the greatest weight were removed (S2 Table). The combined RR of the remaining 7 studies was 0.94 (95% CI: 0.93–0.96, Figure 2), with no significant heterogeneity (Q = 5.52, *P* < 0.479; I^2^ = 0%). Furthermore, in an analysis that included these 2 studies, the combined RR was 0.91 (95% CI: 0.86–0.97), showing consistency with the result above. No potential publication bias was found (Begg *P* = 0.348; Egger *P* = 0.785, Figure 3) for the 9 included studies.

### Association Between Physical Activity and the Risk of Wrist Fracture in Older Women

**FIGURE 4:**
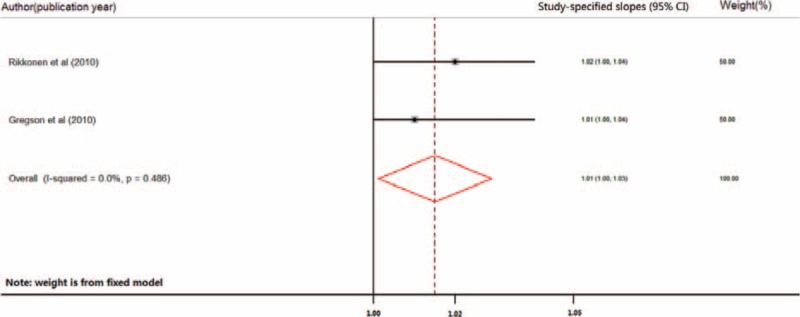
Forest plot (fixed-effect model) of increasing level of physical activity and the risk of wrist fracture of older women. The horizontal line indicates the study-specific 95% confidence interval. The square indicates the study-specific weight from fixed-effect analysis. The diamond indicates the combined relative risk of the 2 included studies after the sensitivity analysis.

Three studies were included in the dose–response meta-analysis for wrist fracture,^13,14,20^ with a combined RR of 1.004 (95% CI: 0.98–1.03) for every increase of 3 physical-activity units. Heterogeneity was significant between the 3 studies (Q = 10.53, *P* < 0.005; I^2^ = 81.0%). The combined RR of the studies remaining after omitting the study by Armstrong et al^20^ was 1.01 (95% CI: 1.00–1.03, Figure 4), with no significant heterogeneity (Q = 0.460, *P* < 0.500; I^2^ = 0%). Egger test and Begg test indicated no significant publication bias between the 3 studies (*P* = 0.296 and *P* = 0.107, respectively).

## DISCUSSION

Physical activity has been proofed to benefit bone health in elder population.^32^ However, exercise may also cause accident fall, sports injury, and even fracture in elder population. Results of several previous studies were inconsistent,^12–17^ and at what level of physical activity would influence the risk of fracture in older woman was uncertain. Thus, we carried out a meta-analysis and found a dose–response relationship between physical activity and risk of hip fracture in elder women. The risk of hip fractures showed an inverse association with increasing levels of physical activity.

As a persistent cause of excessive morbidity, fracture of elder population continues as a serious public health problem and is of high concern. Osteoporosis is considered to be the most common reason for fracture among old people.^33,34^ Older women are more vulnerable to osteoporosis fracture due to increased bone loss after menopause. Bone loss is inevitable due to lower levels of estrogen.^20^ Although increasing physical activity levels resulting in better muscle strength and balance could decrease the risk of osteoporosis fracture, the procedure can delay bone demineralization,^35,36^ and reinforce bone architecture and strength.^37^ Accident fall is another important cause of fracture, especially hip and wrist fracture.^19,38^ Although strenuous activity may result in accident fall and fracture, leisure physical activity is usually hypothesized to reduce the risk of fall and fall-related fracture in multiple ways.^32^On one hand, physical activity participation was related with health status of older people. Lower level of physical activities usually indicates worse health status and susceptibility to fall and fracture.^39^ On the other hand, higher participation of exercise helps to maintain joint flexibility, muscle strength, and balance, connected with decreased risk of accident fall and risk of fracture.^40^ In our study, increasing levels of physical activity reduced the risk of hip fracture. The result is highly consistent with the theoretical hypothesis and studies.

There are several limitations of the study. First, we tried to uniform the dose of physical activity of different studies using standard criteria; however, the assessment was not precise. And only common leisure activity was reported in the included studies. Thus, strenuous exercise was not recommended for elder people. Second, the baseline characteristics of each study were not the same. Although increasing age, lifestyle, and economic conditions are known to be associated with osteoporosis and fractures, whether those factors have similar effects in different cohorts could not be well estimated. Especially, the heterogeneity of baseline age may lead to bias in the study. Third, calcium, Vitamin-D_3_, and other anti-osteoporotic drugs were popularized for long in elder women; it was not considered in several included studies. Thus, even smoking, diabetes, and other covariates were adjusted; the effect of anti-osteoporotic treatment may be an important confounder of the study. The last, the study has a significant ethnic background. The included cohorts were mainly from Europe and the USA, and generalization of the conclusion should be cautious. Further studies are still needed to investigate what kind of physical activity and how best physical activities could reduce the risk of fracture in elder population.

## CONCLUSIONS

Our results showed that increasing level of leisure physical activity reduced the risk of hip fracture, but not the risk of wrist fracture in older women.

## Supplementary Material

Supplemental Digital Content

## Supplementary Material

Supplemental Digital Content
